# Potential Association Between Asthma, *Helicobacter pylori* Infection, and Gastric Cancer

**DOI:** 10.3389/fonc.2021.630235

**Published:** 2021-03-08

**Authors:** Fengxia Wu, Cai Chen, Fulai Peng

**Affiliations:** ^1^School of Basic Medical Sciences, Shandong University, Jinan, China; ^2^Shandong Institute of Advanced Technology, Chinese Academy of Sciences, Jinan, China

**Keywords:** asthma, *Helicobacter pylori* infection, gastric carcinoma, *Coptis chinensis*, network pharmacology, molecular docking

## Abstract

**Background:** The prevalence of *Helicobacter pylori* infection (HPI) is still high around the world, which induces gastric diseases, such as gastric cancer (GC). The epidemiological investigation showed that there was an association between HPI and asthma (AST). Coptidis rhizoma (CR) has been reported as an herbal medicine with anti-inflammatory and anti-bacterial effects.

**Purpose:** The present study was aimed to investigate the protective mechanism of HPI on AST and its adverse effects on the development of GC. *Coptis chinensis* was used to neutralize the damage of HPI in GC and to hopefully intensify certain protective pathways for AST.

**Method:** The information about HPI was obtained from the public database Comparative Toxicogenomics Database (CTD). The related targets in AST and GC were obtained from the public database GeneCards. The ingredients of CR were obtained from the public database Traditional Chinese Medicine Systems Pharmacology (TCMSP). The network pharmacology including gene ontology (GO) enrichment analysis, Kyoto Encyclopedia of Genes and Genomes (KEGG) enrichment analysis, and molecular docking were utilized. Protein–protein interaction was constructed to analyze the functional link of target genes. The molecular docking was employed to study the potential effects of active ingredients from CR on key target genes.

**Result:** The top 10 key targets of HPI for AST were CXCL9, CX3CL1, CCL20, CCL4, PF4, CCL27, C5AR1, PPBP, KNG1, and ADORA1. The GO biological process involved mainly leukocyte migration, which responded to bacterium. The (R)-canadine and quercetin were selected from *C. chinensis*, which were employed to explore if they inhibited the HPI synchronously and protect against AST. The targets of (R)-canadine were SLC6A4 and OPRM1. For ingredient quercetin, the targets were AKR1B1 and VCAM1.

**Conclusion:** CXCL9 and VCAM1 were the common targets of AST and HPI, which might be one of the imported targets of HPI for AST. Quercetin could be an effective ingredient to suppress HPI and help prevent AST.

## Introduction

*Helicobacter pylori* (HP) is a Gram-negative, slightly aerobic bacterium, which attaches to the gastric epithelial cells and requires extremely harsh living conditions (1, 2). Since 1994, the International Agency for Research on Cancer (IARC) had defined HP as a group 1 carcinogen (3). HP infection (HPI) has a high prevalence around the world. It is estimated that in 2015, about 4.4 billion people were with HPI (4). Another survey illustrated that at least 15% Jordanian children were infected by HP (5). One recent study showed that among 350 participants in United Arab Emirates, about 41% were found to be HP infected according to the stool antigen test (6), China, particularly, still faces high prevalence. According to one review about the prevalence of HP published in 2015, the epidemiology of HPI in adults ranged from 41.35 to 72.3%, and it varied with the population studied and with the geographic area (7).

A lot of research has proved that HPI could cause a series of gastrointestinal diseases, such as gastric cancer (GC) with symptoms of nausea, vomiting, upper abdominal discomfort, and dull pain (8–10). Each year, ~990,000 people are diagnosed with GC in the world, of whom about 738,000 died because of GC (11). GC has become the fourth most common incident cancer and the second most common cause of cancer death. As many studies have reported, there is an adverse association between HPI and GC. Tran's research involved 282 patients with non-cardiac GC from Ho Chi Minh and Hanoi, Vietnam, provided the direct evidence that HPI could increase the risk of GC with OR = 2.02 (95% CI: 1.4–3.0) (12). Previous studies have shown that among the long-term consequences of HP is gastric malignancies, particularly GC (13). It was found that the HP cytotoxin-associated antigen A is the major oncogenic factor, which was injected into the host cells and could disrupt epithelial cell functions (14), while the specific pathogenesis of GC caused by HPI has not been figured out.

Asthma (AST) is a heterogeneous disease with the characteristic of chronic airway inflammation involving a variety of cells and cellular components (15). The chronic inflammation is associated with airway hyperreactivity and usually involves extensive and variable reversible restriction of expiratory flow, resulting in recurrent episodes of wheezing, shortness of breath, chest tightness, and cough. AST has become one of the most rapidly growing disorder, which has victimized about one-third of the world's population, and about 2.5 million patients die annually as a result of severe exacerbation (16). According to the epidemiology of AST in the United States, 8.4% of the country's population has AST, and the average annual AST prevalence for children and adults was 9.5 and 7.7%, respectively.

However, one extremely interesting finding was that an epidemiological study found that there was an inverse relationship between HPI and the morbidity of AST. A cross-sectional study showed that the HPI among people, whose age group was <40 years, was inversely correlated with AST (OR, 0.503; 95% CI, 0.280–0.904, *p* = 0.021) (17). Arnold et al. provided evidence *via* animal experiment that there was an association between HPI and GC (18). The HPI is one of the digestive tract/gastric diseases, whereas AST is one of the respiratory diseases, and it is much more interesting that the protective mechanism HP induced for AST and how it connected the respiratory system with the digestive system. *Coptis chinensis* has been reported as an herbal medicine at home and abroad, with anti-inflammatory and anti-bacterial effects (19, 20). As an herbal medicine, it has been widely employed to treat GC, AST, and HPI (21–24).

**Figure 1:**
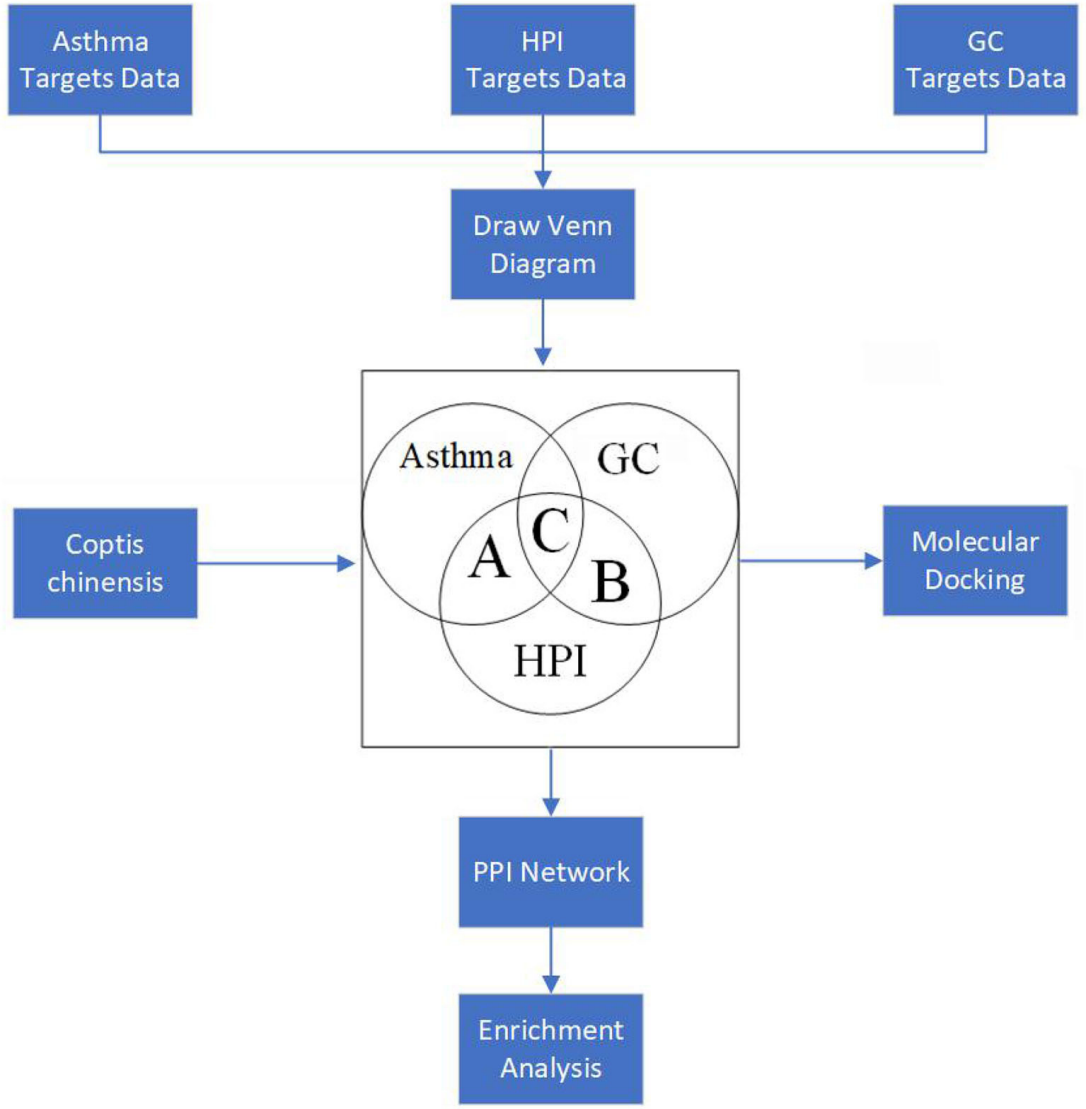
Flowchart of the experimental process (HPI, *Helicobacter pylori* infection; GC, gastric cancinoma; PPI, protein–protein interaction).

In this research, the HPI was considered as a two-sided “drug,” which could cause the gastric discomfort/symptom of digestive tract on one side, such as GC, and on the other side, it could activate certain pathways, which appear protective for AST. Based on the abovementioned opinion, the network pharmacology theory was employed to investigate the relationship between GC, AST, and HPI. The flowchart of the entire paper is illustrated in Figure 1. After searching the common targets for AST, GC, and HPI, *C. chinensis*, selected as a representative drug, was used to neutralize the damage of HPI for GC and to hopefully intensify certain protective pathways for AST. Thus, the creative points of this study are as follows: (1) HPI was assumed as one “drug,” which could damage the stomach and activate the protective pathway for AST. (2) *C. chinensis*, selected as one representative drug, was given hope to compromise the damage caused by HPI.

## Methods

### Data Sources

The related targets about HPI were obtained from public database Comparative Toxicogenomics Database (http://ctdbase.org/) (25), where it could provide relationships in the human disease hierarchy and detailed information, including associated chemicals and genes. The related targets in AST and GC were attained from the open database GeneCards (https://www.genecards.org/) (26), which integrates gene-centric data from more than 150 web sources, including genomic, transcriptomic, proteomic, genetic, clinical, and functional information. The relevance score was set at more than 5, considering that the weakly related targets could distract the subsequent main network.

### Protein–Protein Interaction Networks Construction

The targets of HP, GC, and AST were intersected *via* the tool Draw Venn Diagrams (http://bioinformatics.psb.ugent.be/webtools/Venn/) (27), which is a tool that could calculate the intersection of input elements and give the Venn map. It was used to select the combined targets in this paper. The intersection targets between AST and HP were input into the database String [protein–protein interaction (PPI) networks, https://string-db.org/] with the selecting of multiple proteins and *Homo sapiens*. In its setting section, the minimum required interaction score was set at 0.4, and among display simplifications, the choice of hidden disconnected nodes in the network was selected in order to remove these outliers.

### Enrichment Analysis

After the abovementioned processes, the network for AST and HPI was constructed successfully for the sake of distinction, and it was named as AST–HPI network in this paper. Then, AST–HPI network was input into the software Cytoscape for visualization (28) (https://cytoscape.org/). For interaction targets of GC and HP, it was performed with the same abovementioned operation, and its network was called GC–HPI network. Gene ontology (GO) and Kyoto Encyclopedia of Genes and Genomes (KEGG) enrichment analyses were operated on Metascape (https://metascape.org/gp/index.html#/main/step1). After intersection targets were imported into Metascape, *H. sapiens* and custom analysis were selected to calculate the GO cellular components, biological processes, molecular functions, and KEGG pathway.

The ingredients of coptidis rhizoma (CR) were obtained from the public database Traditional Chinese Medicine Systems Pharmacology (https://tcmspw.com/tcmsp.php), which can provide the related information, including ingredients name, oral bioavailability, drug-likeness, etc. In order to filter the main components, the drug-likeness and oral bioavailability were >0.18 and 30, respectively. Then, corresponding targets were collected in this database. The intersection dataset between HPI and CR was calculated *via* the Draw Venn Diagrams, and the PPI networks of CR and HP were established by String as described earlier. Here, the targets for the potential valid components of CR were discovered. AutoDock Vina (http://vina.scripps.edu/) was implemented to molecular docking to study the effects on key target genes.

## Results

### Target Selection

**Figure 2:**
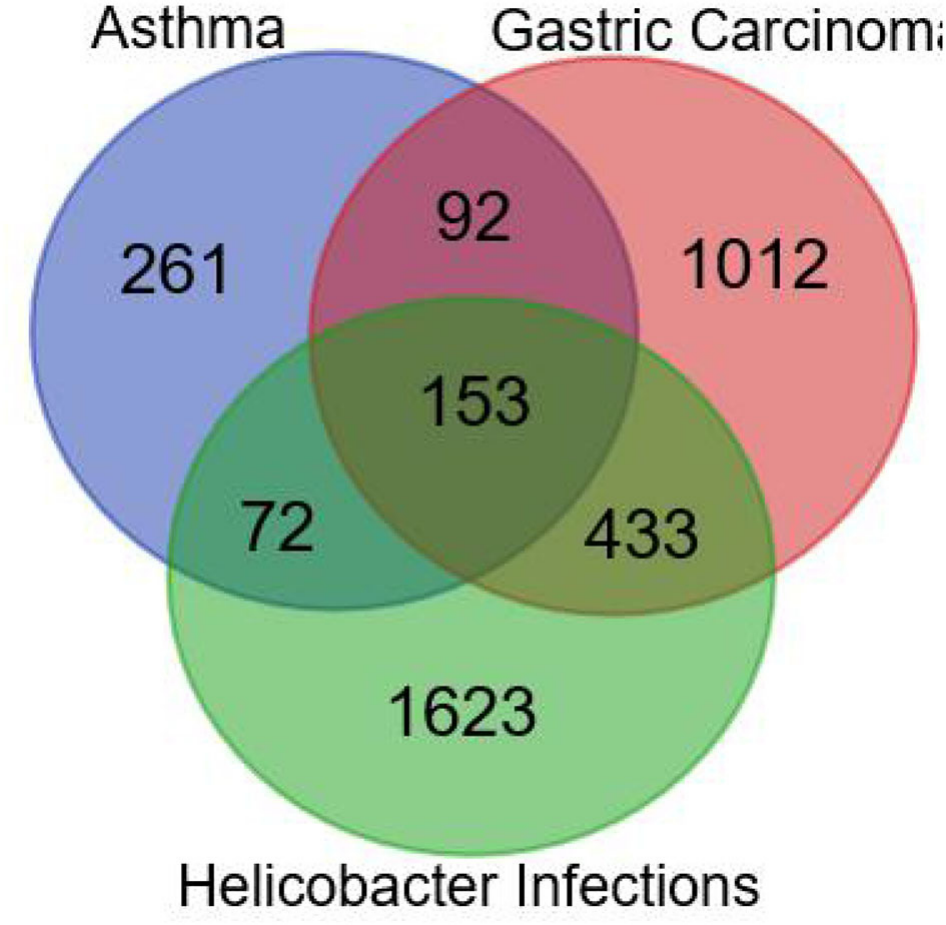
Combined target for asthma, gastric cancer, and *Helicobacter pylori* infection.

As shown in Figure 2, the number of the targets of HPI, AST, and GC were 2,281, 578, and 1,690, respectively. The number of targets for AST and HPI not containing GC was 72, which was corresponding to section A in Figure 1. The number of GC and HPI not containing AST was 433, which was corresponding to section B in Figure 1. The number of intersection dataset of them was 153, as opposed to section C in Figure 1.

### Protein–Protein Interaction Network Construction Based on Section A

There were 72 interaction nodes (targets) and 308 edges according to the PPI network constructed in String, and the *p*-value of PPI network was <1.0^e−16^, which was statistically significant. The 72 common targets contain VCAM1, FGA, SYK, CX3CL1, CXCL9, TNFRSF1B, OPRM1, SRSF2, IL16, LTA, CCL3, and others.

**Figure 3:**
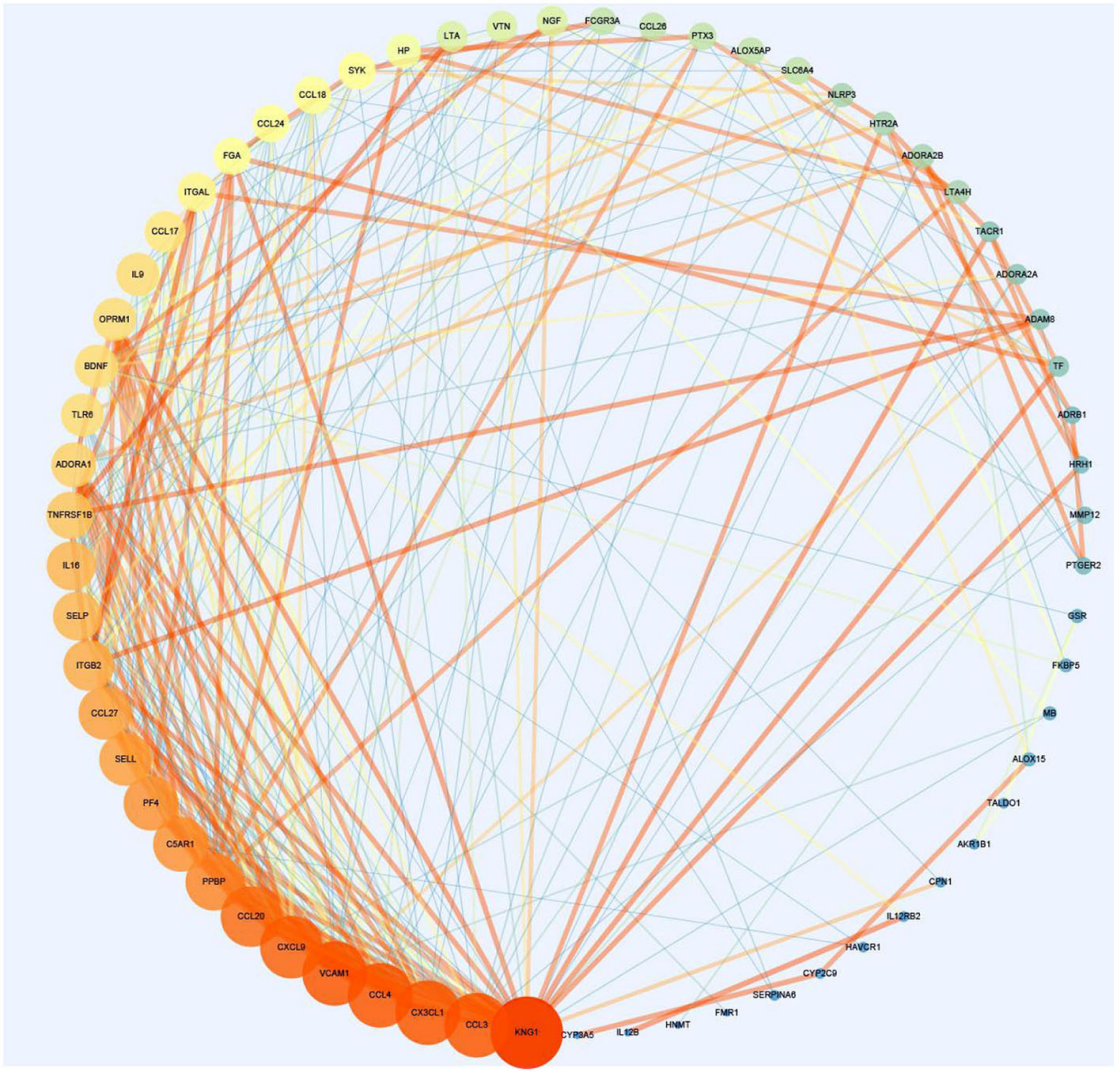
The protein–protein interaction network for asthma and *Helicobacter pylori* infection (section A).

**Figure 4:**
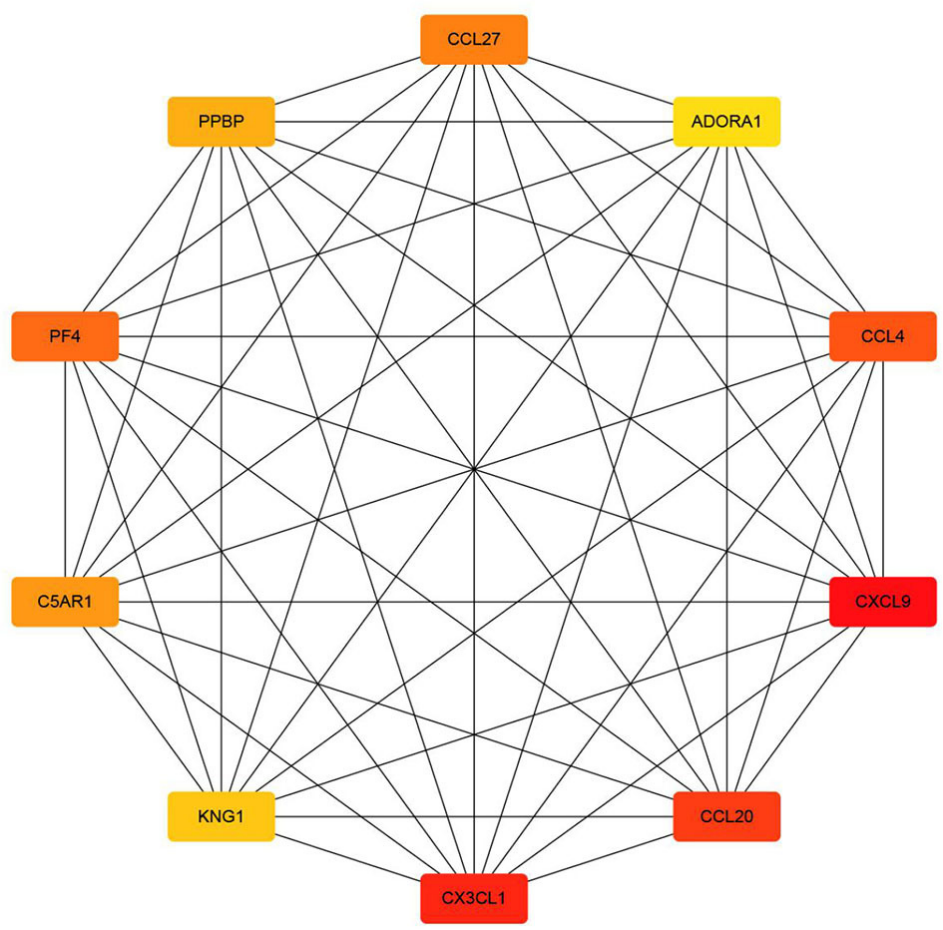
The top 10 key targets (section A).

Protein–protein interaction network is shown in Figure 3. Degrees of freedom (Df), one of the topology parameters in this network, represented the number of connections between different nodes, which were illustrated by the size of the nodes. KNG1 had the highest Df, which means that it possessed the most connection nodes. Another topology parameter, node closeness is the degree of closeness for this node and other nodes, which was expressed by the color of the node. The deeper the color, the greater the correlation. Combined score represents the correlation strength between 2 nodes, which was displayed by the thickness of the edge. The thicker the edge, the stronger the correlation. The average of Df and the average clustering coefficient were 8.56 and 0.532, respectively. Using cytoHubba in Cytoscape, the top 10 key targets were CXCL9, CX3CL1, CCL20, CCL4, PF4, CCL27, C5AR1, PPBP, KNG1, and ADORA1 (Figure 4).

**Figure 5:**
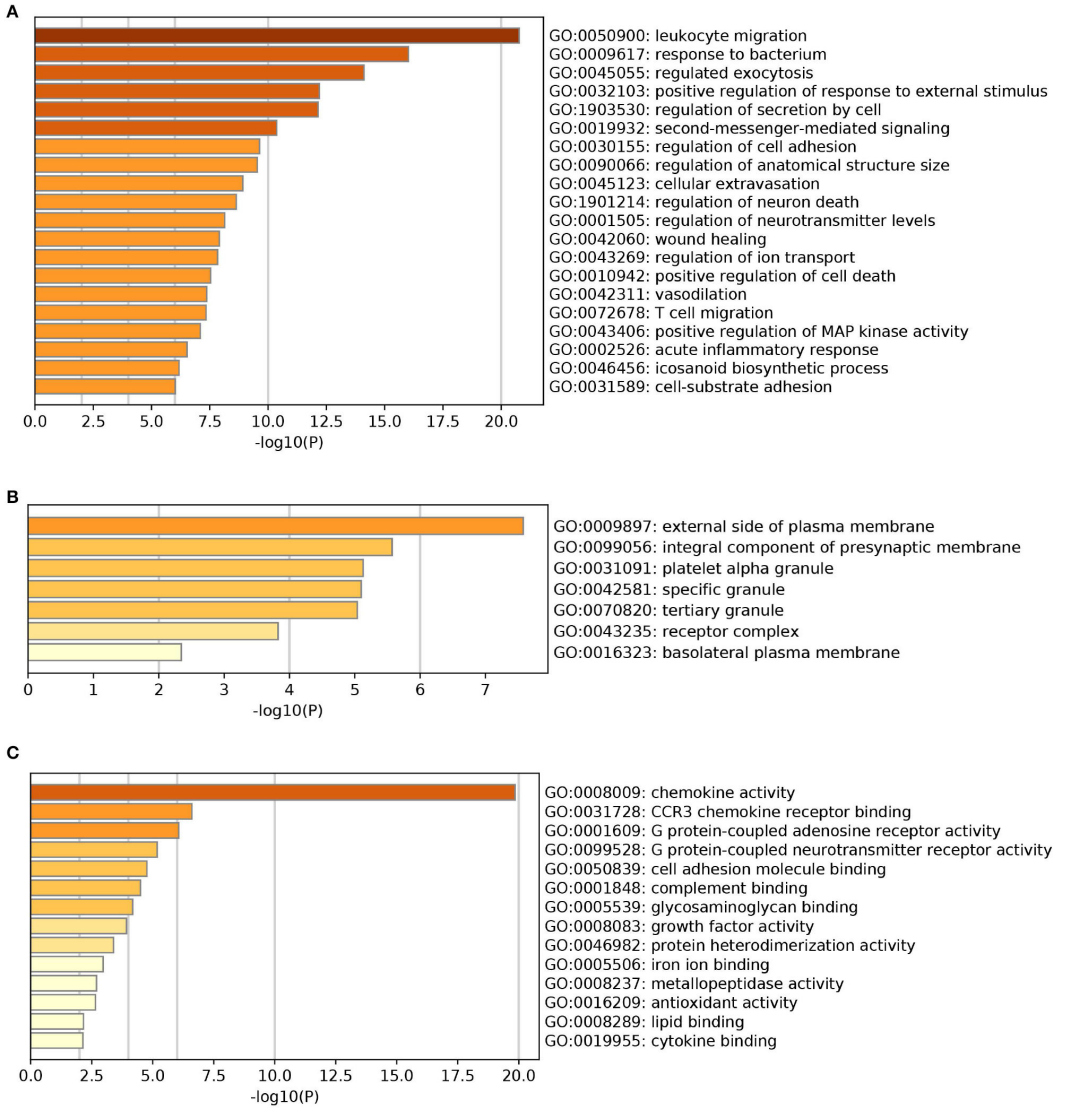
The gene ontology (GO) enrichment analysis of the interaction targets (section A) for asthma and *Helicobacter pylori* infection [**(A)** biological process; **(B)** cellular component; and **(C)** molecular function].

**Figure 6:**
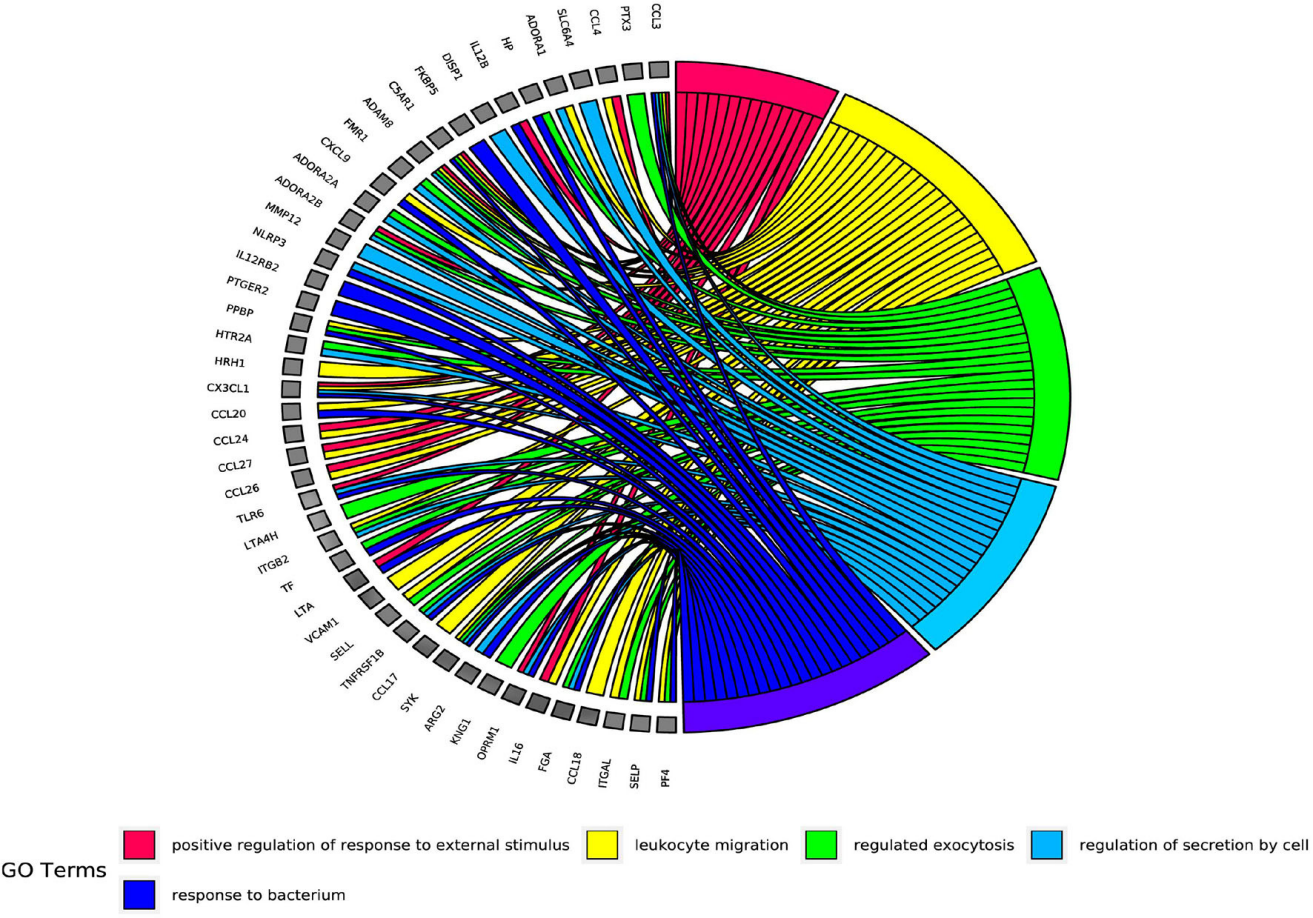
The gene ontology (GO) chord plot of top 5 ranked overrepresented GO terms belonging to the biological process.

**Figure 7:**
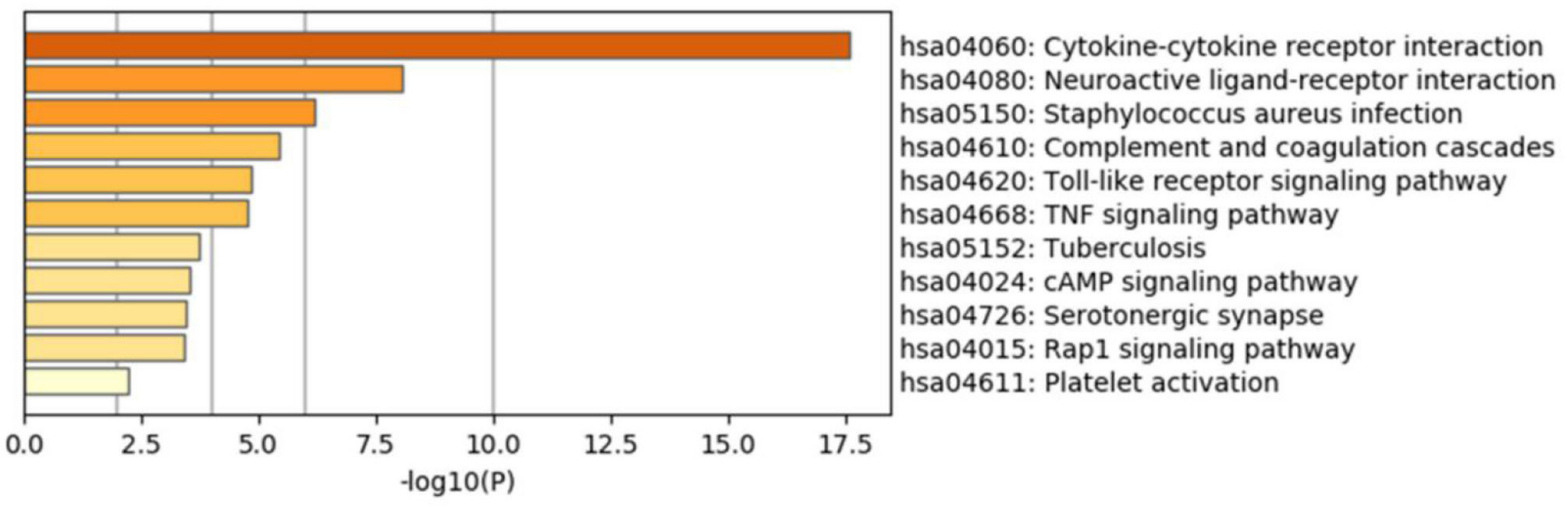
Enrichment analysis results of the Kyoto Encyclopedia of Genes and Genomes (KEGG) signaling pathway for section A.

The results of GO enrichment analysis *via* Metascape are illustrated in Figure 5 and Tables 1–3. The GO biological process mainly included leukocyte migration, response to bacterium, regulated exocytosis, positive regulation of response to external stimulus, etc. The GO cellular components mainly included external side of plasma membrane, integral component of presynaptic membrane, platelet alpha granule, specific granule, etc. For molecular functions process, it mainly included chemokine activity, CCR3 chemokine receptor binding, G protein-coupled adenosine receptor activity, G protein-coupled neurotransmitter receptor activity, and cell adhesion molecule binding. The GO chord plot of the top 5 ranked overrepresented GO terms belonging to the biological process is displayed in Figure 6. The enrichment analysis results of KEGG signaling pathway for section A are presented in Figure 7 and Table 4. It was apparent that *Staphylococcus aureus* infection, toll-like receptor signaling pathway, tuberculosis, and Rap1 signaling pathway were involved in this process.

**Table 1:** GO enrichment analysis of molecular functions.

**GO term**	**Description**	**Count**	**Log_**10**_(P)**
GO:0008009	Chemokine activity	12	−19.85
GO:0031728	CCR3 chemokine receptor binding	3	−6.61
GO:0001609	G protein-coupled adenosine receptor activity	3	−6.07
GO:0099528	G protein-coupled neurotransmitter receptor activity	4	−5.18
GO:0050839	Cell adhesion molecule binding	9	−4.76

**Table 2:** GO enrichment analysis of cellular components.

**GO term**	**Description**	**Count**	**Log_**10**_(P)**
GO:0009897	External side of plasma membrane	11	−7.58
GO:0099056	Integral component of presynaptic membrane	5	−5.57
GO:0031091	Platelet alpha granule	5	−5.13
GO:0042581	Specific granule	6	−5.1
GO:0070820	Tertiary granule	6	−5.04
GO:0043235	Receptor complex	8	−3.83
GO:0016323	Basolateral plasma membrane	4	−2.34

**Table 3:** GO enrichment analysis of biological processes.

**GO term**	**Description**	**Count**	**Log_**10**_(P)**
GO:0050900	Leukocyte migration	23	−20.77
GO:0009617	Response to bacterium	22	−16.02
GO:0045055	Regulated exocytosis	21	−14.11
GO:0032103	Positive regulation of response to external stimulus	14	−12.2
GO:1903530	Regulation of secretion by cell	19	−12.14

**Table 4:** Enrichment analysis of KEGG signaling pathway of HPI-AST network.

**GO**	**Description**	**Count**	**Log_**10**_(P)**
hsa04060	Cytokine-cytokine receptor interaction	17	−17.57
hsa04080	Neuroactive ligand-receptor interaction	10	−8.05
hsa05150	*Staphylococcus aureus* infection	5	−6.18
hsa04610	Complement and coagulation cascades	5	−5.43
hsa04620	Toll-like receptor signaling pathway	5	−4.84
hsa04668	TNF signaling pathway	5	−4.76
hsa05152	Tuberculosis	5	−3.72
hsa04024	cAMP signaling pathway	5	−3.52
hsa04726	Serotonergic synapse	4	−3.45
hsa04015	Rap1 signaling pathway	5	−3.4
hsa04611	Platelet activation	3	−2.24

### Protein–Protein Interaction Network Construction Based on Section B

**Figure 8:**
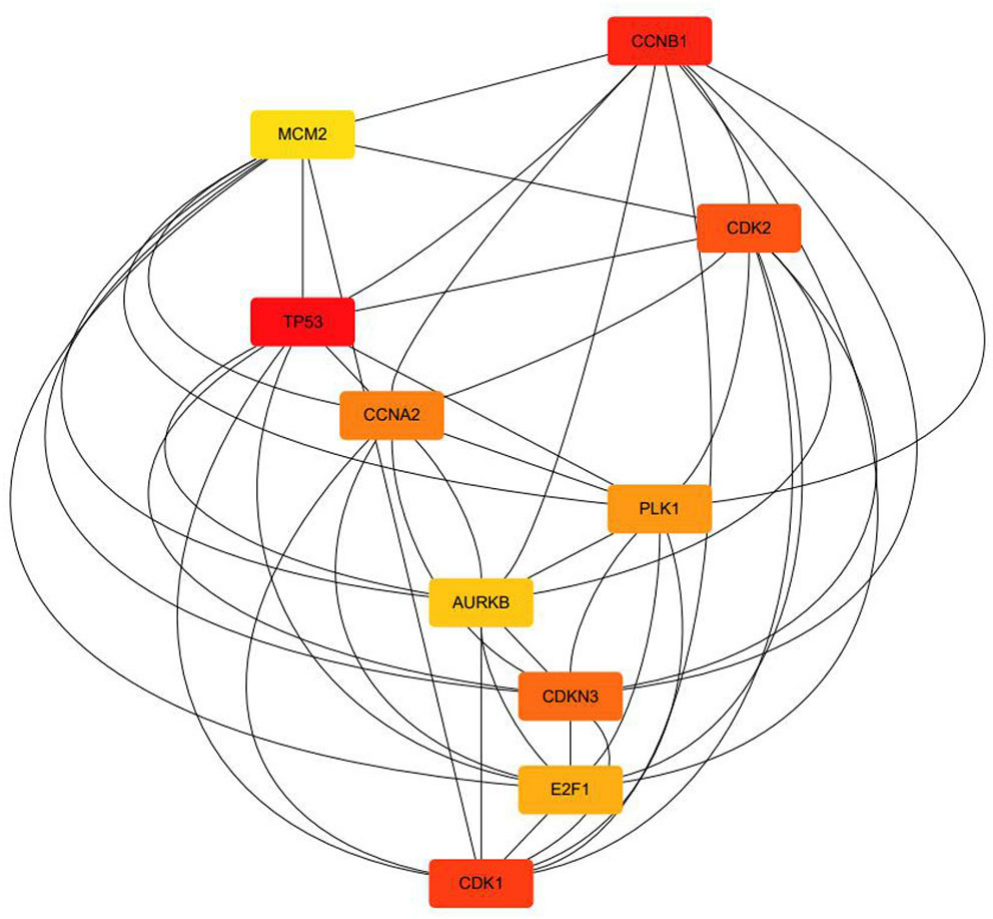
The top 10 key targets (section B).

**Figure 9:**
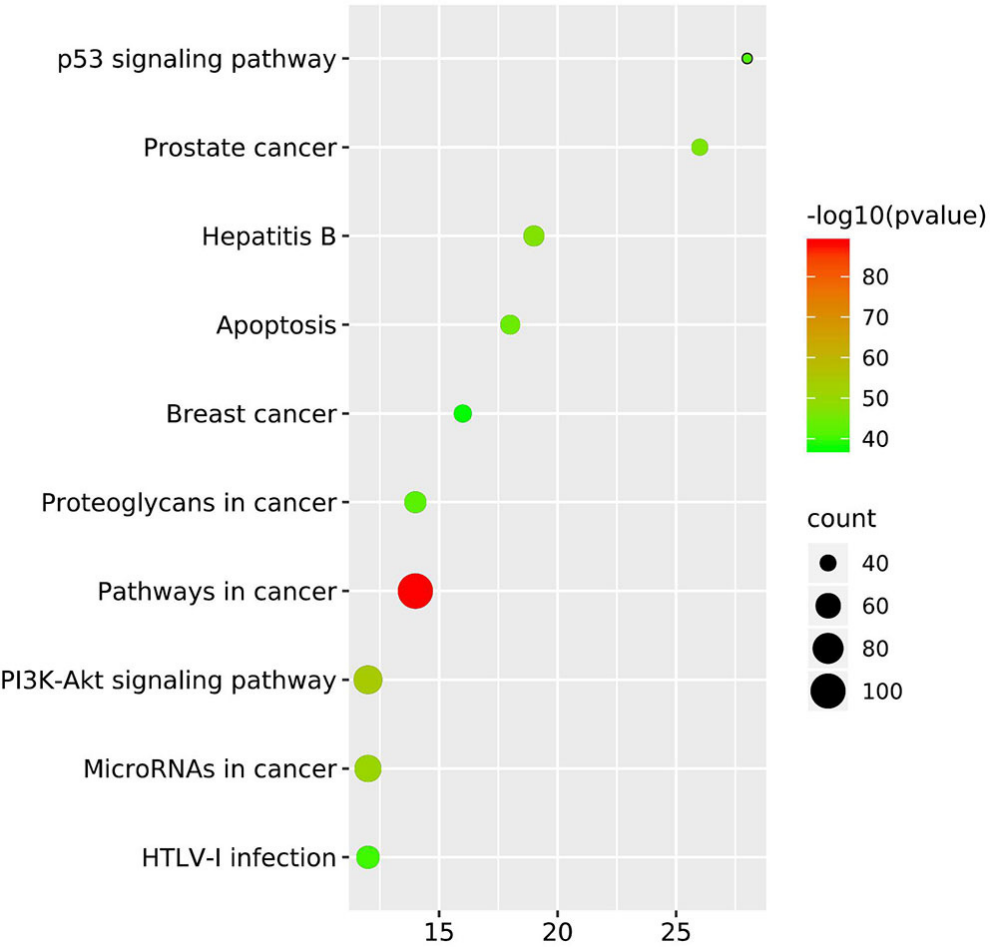
The Kyoto Encyclopedia of Genes and Genomes (KEGG) enrichment pathway result.

The top 10 key targets are presented in Figure 8. They are CCNB1, MCM2, CDK2, TP53, CCNA2, PLK1, AURKB, CDKN3, E2F1, and CDK1, respectively. The KEGG pathway enrichment analysis result based on Metascape are displayed in Figure 9, which mainly consisted of p53 signaling pathway, prostate cancer, hepatitis B, apoptosis, breast cancer, proteoglycans in cancer, pathways in cancer, PI3K-Akt signaling pathway, microRNAs in cancer, and Human T lymphocyte leukemia virus (HTLV)-l infection.

**Figure 10:**
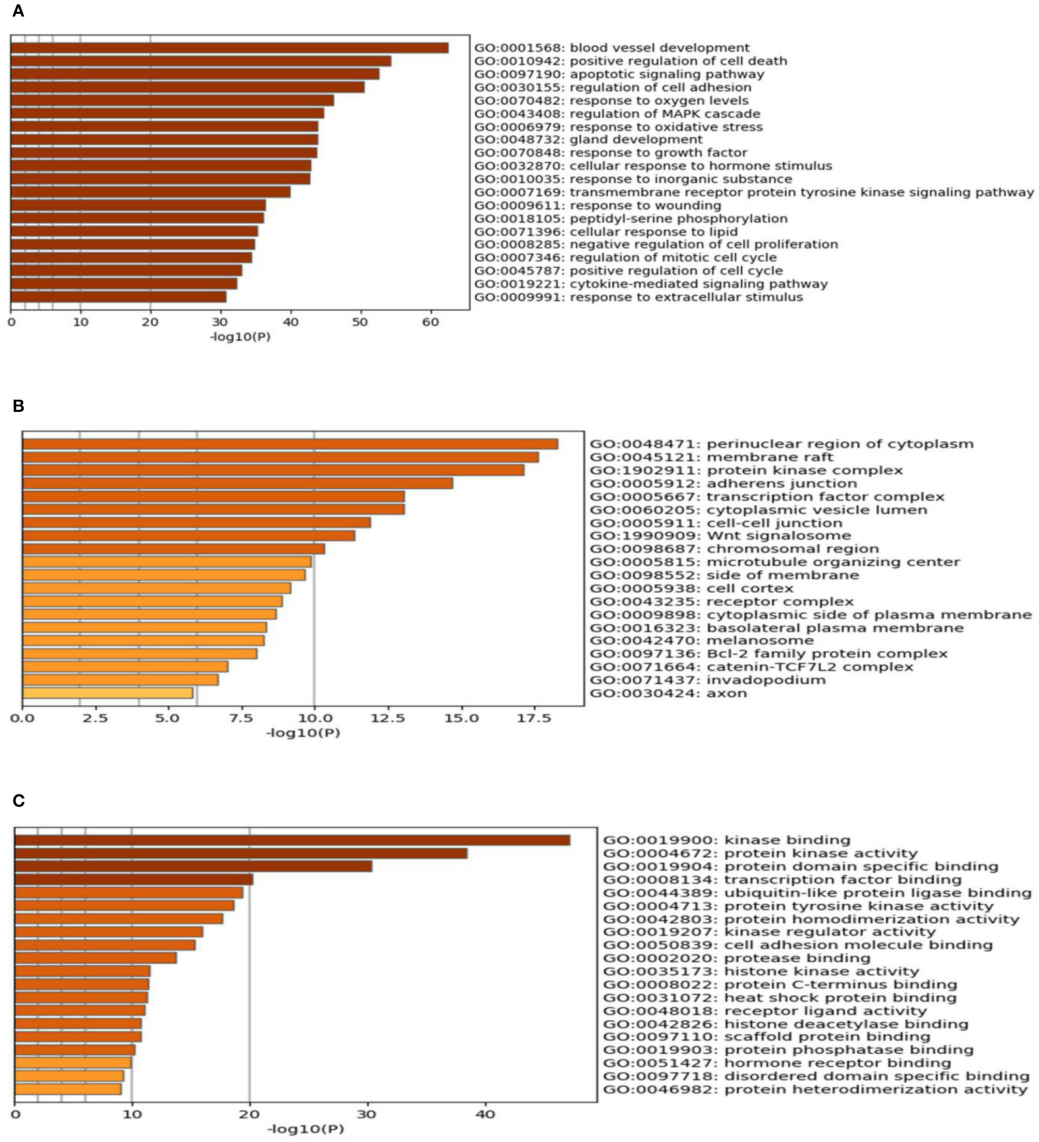
The gene ontology (GO) enrichment analysis of the interaction targets (section B) for gastric carcinoma and *Helicobacter pylori* infection [**(A)** biological process; **(B)** cellular component; and **(C)** molecular function).

The results of the GO enrichment analysis are illustrated in Table 5 and Figure 10. The GO biological processes that were involved mainly consisted of positive regulation of cell death, apoptotic signaling pathway, response to oxygen levels, blood vessel development, and so on. For cellular components, it mainly contained adherens junction, perinuclear region of cytoplasm, protein kinase complex and transcription factor complex, and so on. According to Figure 10C, it is found that kinase binding, protein kinase activity, protein domain specific binding, transcription factor binding, and ubiquitin-like protein ligase binding participated in the process. Section C contained totally 153 key targets as shown in Table 6, including CD40, CXCR1, IL10, IL6, etc.

**Table 5:** The part result of GO enrichment analysis of section B.

**GO**	**Category**	**Description**	**Count**	**Log_**10**_(P)**
GO:0001568	Biological processes	Blood vessel development	106	−62.44
GO:0010942	Biological processes	Positive regulation of cell death	96	−54.23
GO:0097190	Biological processes	Apoptotic signaling pathway	87	−52.48
GO:0030155	Biological processes	Regulation of cell adhesion	91	−50.42
GO:0070482	Biological processes	Response to oxygen levels	68	−46.09
GO:0048471	Cellular components	Perinuclear region of cytoplasm	54	−18.27
GO:0045121	Cellular components	Membrane raft	36	−17.6
GO:1902911	Cellular components	Protein kinase complex	22	−17.13
GO:0005912	Cellular components	Adherens junction	42	−14.69
GO:0005667	Cellular components	Transcription factor complex	32	−13.05
GO:0019900	Molecular functions	Kinase binding	89	−47.18
GO:0004672	Molecular functions	Protein kinase activity	73	−38.46
GO:0019904	Molecular functions	Protein domain specific binding	69	−30.31
GO:0008134	GO molecular functions	Transcription factor binding	54	−20.2
GO:0044389	GO molecular functions	Ubiquitin-Like protein ligase binding	37	−19.37

**Table 6:** Intersection dataset for asthma, *Helicobecter pylori*, and gastric cancer.

CD44,MMP2,EDN1,CXCR4,EP300,NOS2,TLR1,COL1A1,NFKB2,TNFRSF10A,GHRL,IL6RCRP,GSTP1,STAT5A,NFE2L2,BMP6,HRH2,TNF,EGF,IL1A, GNRH1,CREBBP,KIT,JAK2,CASP1,F2,PTGS2,IL18,NPY,TAC1,SST,VEGFA, AREG,TGFB1,MME,NTS,SOCS3,TIMP1,GAL,MMP1,STAT1,CD14,IDO1,HMOX1,INS,MMP3,GATA3,CXCR1,CRH,IL10,MAPK1,POSTN,EGFR,SOD1, CREB1,LGALS3,IL4,G6PD,GZMB,HLA-G,IL1RN,TGFBR1,IL3,S100A8, CXCL10,IL4R,CAT,IL1B,POMC,NFKBIA,IGFBP3,ALB,NOD2,GAPDH,TLR2,SERPINA3, IVL,KLK3,CXCL8,EGR1,SELE,ABCB1,NR3C1,THBD,PIK3C2A, MAPK14,PLA2G4A,DRD2,MPO,ELN,CSF3,STAT3,ARG1,IL2,IFNG,CCL2,IL6, VDR,ACE,ITGAM,FASLG,KRT14,IL2RA,CD80,NOD1,NQO1,SP1
COMT,HMGB1,MIR146A,TGFB2,PLAU,CD4,IRF1,ALOX5,NOS1,PRL,SMAD3,PLA2G2A,FAS,CSF1,TNFSF10,CXCR2,CD40,CASP8,PPARG,CCL7,CALCA,SERPINA1,HLA-DQB1,CXCL12,ICAM1, MAPK3, IL6ST, CXCL1, CFTR,IFNA1, HLA-DRB1, HLA-B, CXCL5,CCR5,MIF,KITLG,PTGS1,F3, JUN,CP,GSTT1, CYP3A4, S100A9, CSF2,MMP9

### Network Pharmacology Analysis

**Figure 11:**
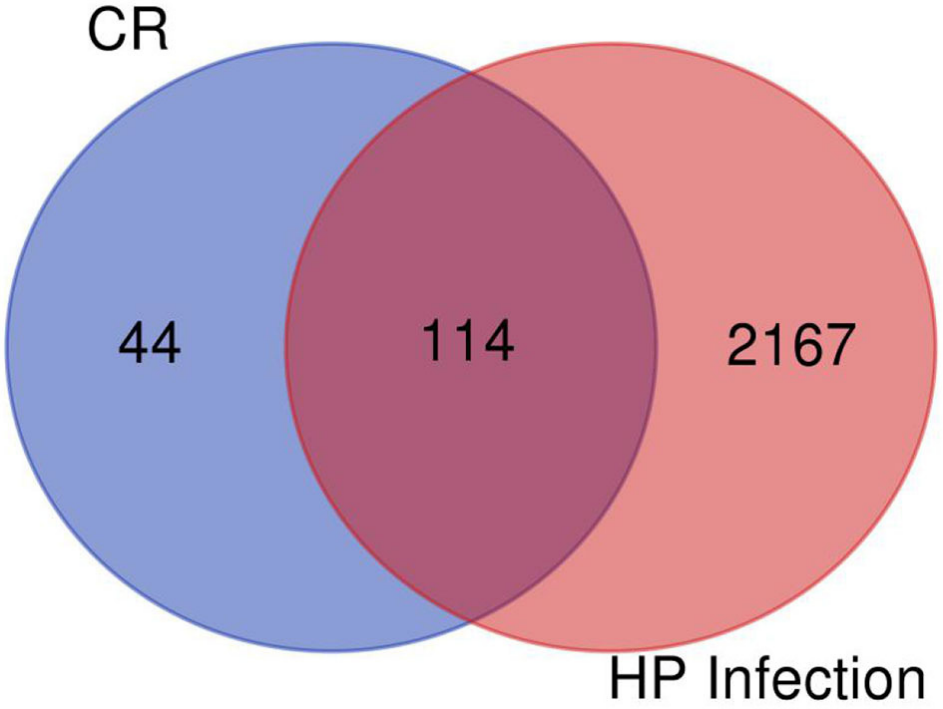
The intersection dataset between *Helicobacter pylori* infection (HPI) and coptidis rhizoma (CR).

After filtering, there were 14 ingredients from CR in this study, as presented in Table 7. About 288 targets of these ingredients were provided in Supplementary Materials. The intersection dataset between HPI and CR *via* Draw Venn Diagrams contains 114 elements as shown in Figure 11. According to the network *via* String, there are 4 targets of ingredients of CR, which belongs to section A (Table 8). It was found that the targets of (R)-canadine were SLC6A4 and OPRM1. For ingredients of quercetin, the targets were AKR1B1 and VCAM1. It was found that NOS2 is the common target of coptisine, berberrubine, berlambine, and berberine (Table 9).

**Table 7:** Components of coptidis rhizoma involved in this study.

	**Mol ID**	**Molecule name**	**Oral bioavailability (%)**	**Drug-Likeness**
1	MOL001458	Coptisine	30.67	0.86
2	MOL000762	Palmidin A	35.36	0.65
3	MOL002894	Berberrubine	35.74	0.73
4	MOL002904	Berlambine	36.68	0.82
5	MOL001454	Berberine	36.86	0.78
6	MOL002897	Epiberberine	43.09	0.78
7	MOL013352	Obacunone	43.29	0.77
8	MOL002668	Worenine	45.83	0.87
9	MOL000098	Quercetin	46.43	0.28
10	MOL002903	(R)-Canadine	55.37	0.77
11	MOL000622	Magnograndiolide	63.71	0.19
12	MOL000785	Palmatine	64.6	0.65
13	MOL008647	Moupinamide	86.71	0.26
14	MOL002907	Corchoroside A_qt	104.95	0.78

**Table 8:** Ingredient-Target existing in section A.

**Ingredient**	**Target**	**Description**
(R)-Canadine	SLC6A4	Solute carrier family 6 member 4
(R)-Canadine	OPRM1	Opioid receptor Mu 1
Quercetin	AKR1B1	Aldo-Keto reductase family 1 member B
Quercetin	VCAM1	Vascular cell adhesion molecule 1

**Table 9:** Ingredient-Target existing in section B.

	**Ingredient**	**Target**
1	Coptisine	NOS2; PTGS1; AR; PTGS2
2	Berberrubine	NOS2; PTGS1; AR; PTGS2
3	Berlambine	NOS2; PTGS1; AR; PTGS2
4	Berberine	NOS2; PTGS1; AR; PTGS2
5	Epiberberine	NOS2; AR; PTGS2
6	Worenine	NOS2; PTGS1; AR; PTGS2; CHEK1
7	(R)-Canadine	PTGS1; PTGS2
8	Quercetin	BCL2; BCL2L1; FOS; CDKN1A; CASP3; ODC1; RAF1; HIF1A; RUNX1T1; PPARG; CYP1A2; MYC; CYP1A1; PRKCB; TOP2A; AHR; CHEK2; HSF1; SPP1; RUNX2; E2F1; IGF2
9	Magnograndiolide	GABRA1
10	Palmatine	NOS2
11	Corchoroside A_qt	NR3C2

**Figure 12:**
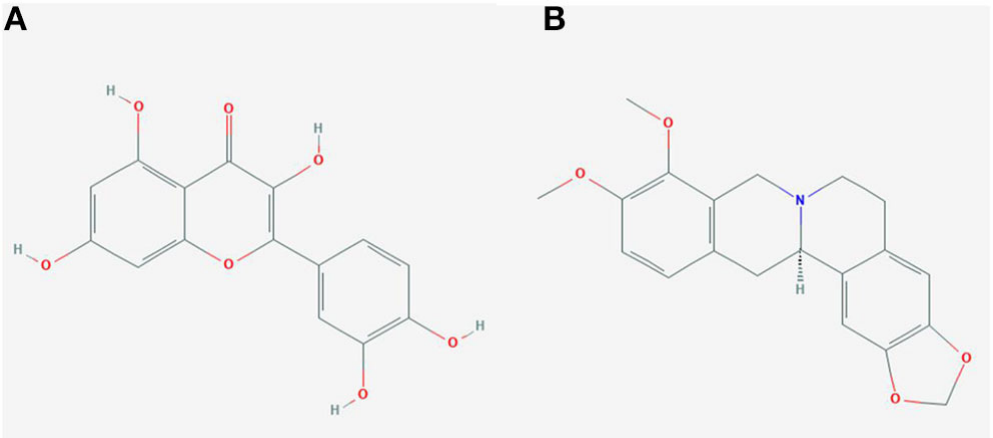
The second structure of quercetin **(A)** and (R)-canadine **(B)**.

**Figure 13:**
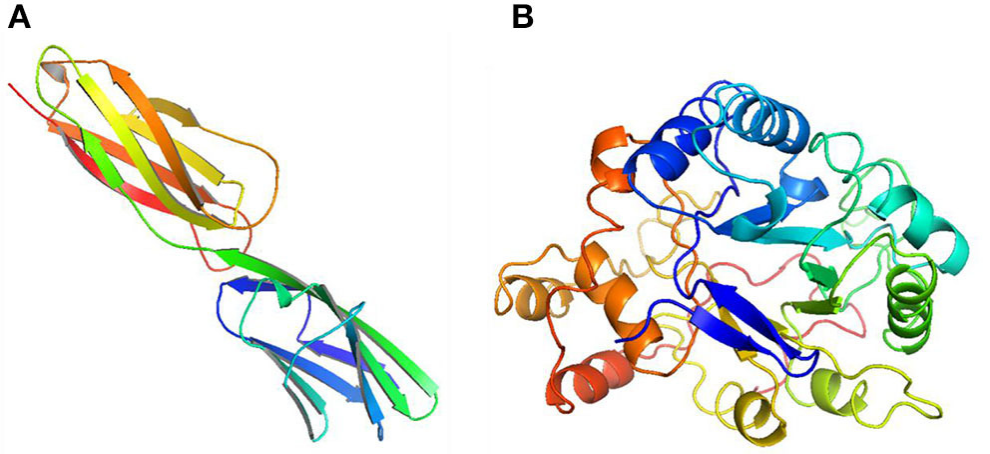
The three-dimensional structure of VCAM1 **(A)** and AKR1B1 **(B)**.

**Figure 14:**
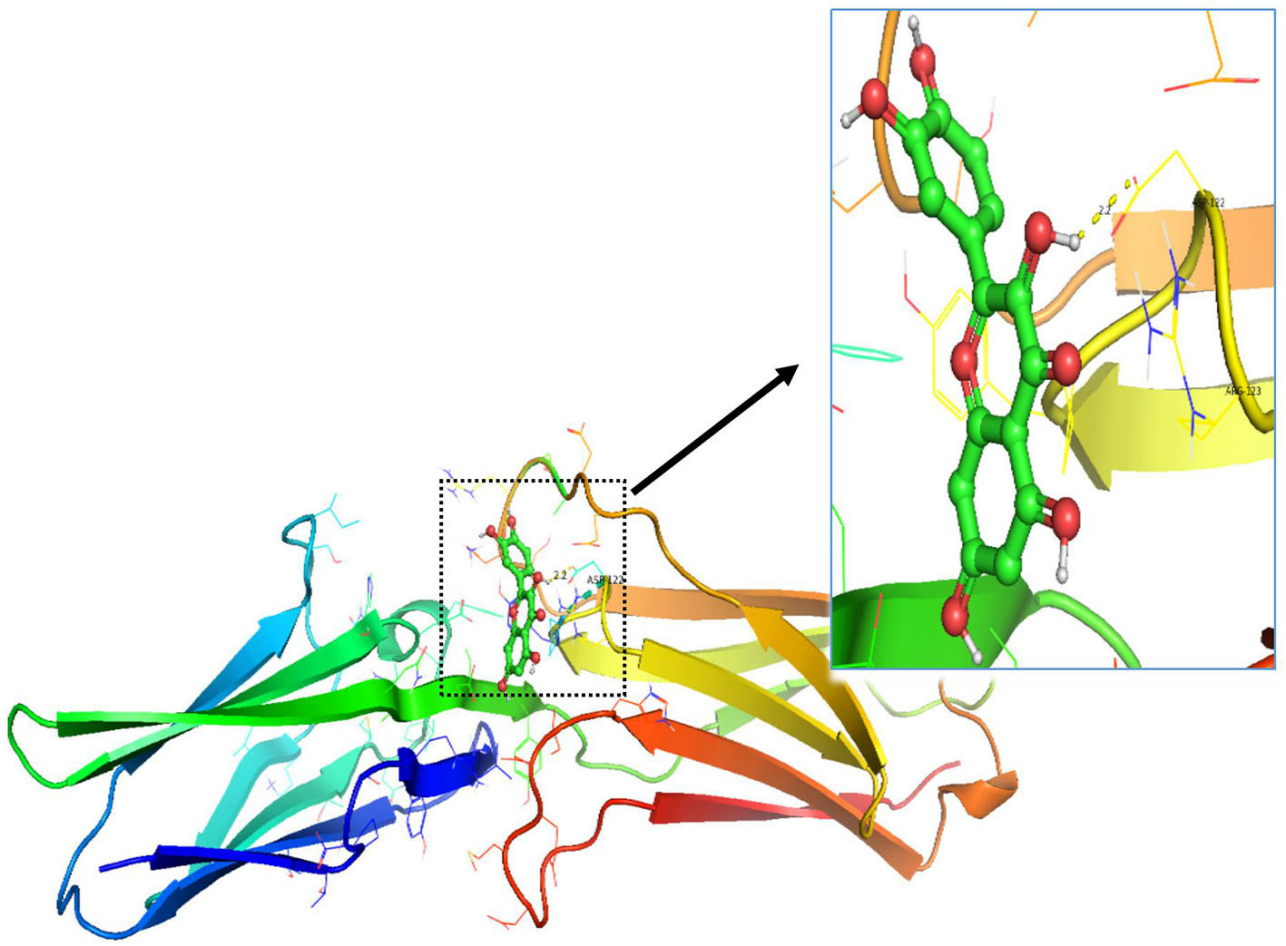
The result of molecular docking between quercetin and VCAM1.

**Figure 15:**
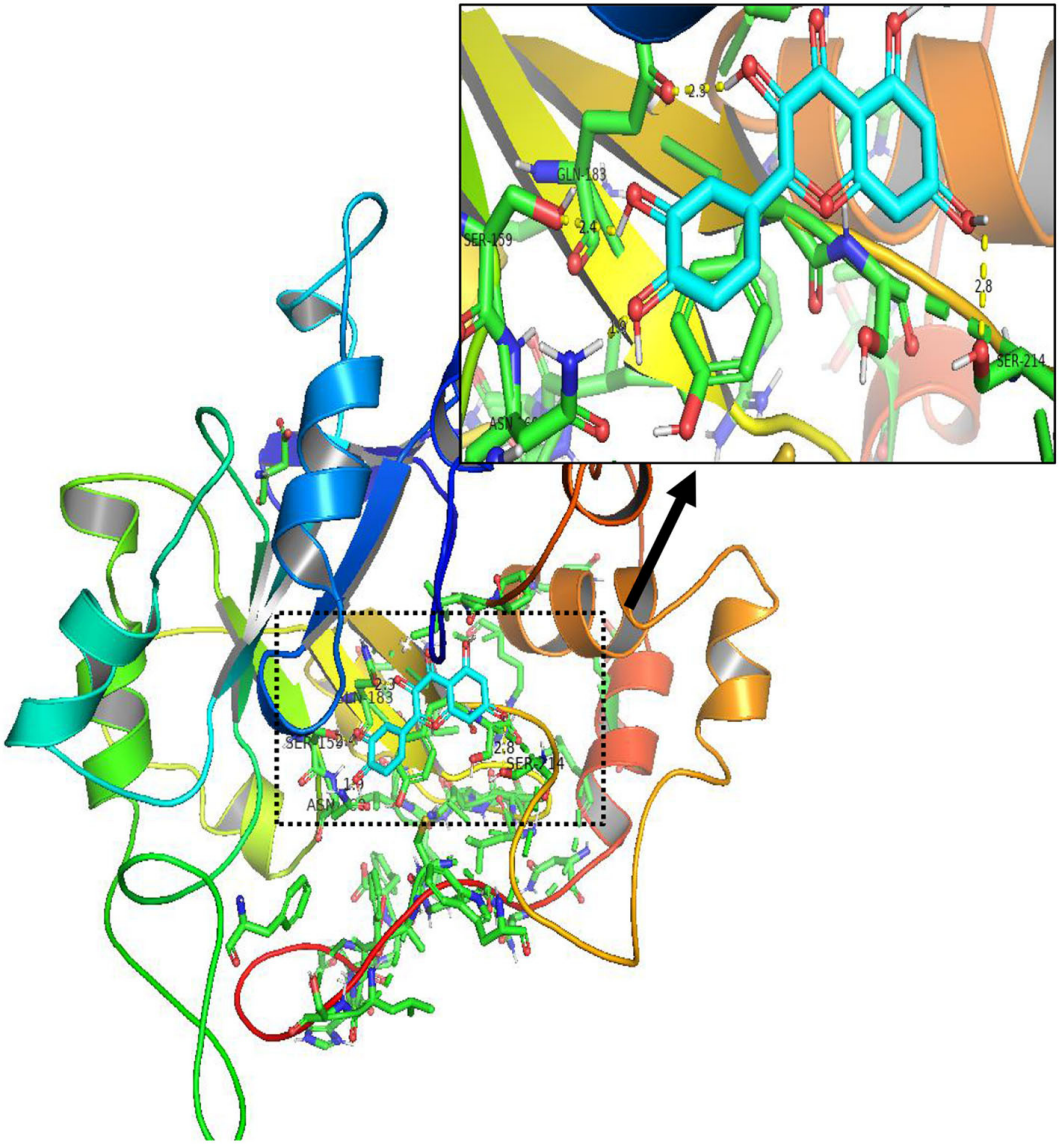
The result of molecular docking between quercetin and AKR1B1.

In order to test the probability of the combination of target and ingredient, molecular docking between quercetin and VCAM1, and also between quercetin and AKR1B1, was performed. Second structure of quercetin from PubChem was transformed to three-dimensional structure *via* Chem3D with the operation of energy minimization (Figure 12). Three-dimensional structure of VCAM1 and AKR1B1 was obtained from protein data bank (PDB) (Figure 13). The molecular visualization software Pymol (https://pymol.org/2/) was used to dehydrate the receptor (by using command: remove solvent), remove unwanted heteroatoms (by using command: remove organic), and save it as the receptor structure. The molecular docking was operated in AutoDock Vina after the relevant configuration, and the result is as shown in Figures 14, 15. There was one junction point for the result of molecular docking between quercetin and VCAM1, and its bond strength is 2.2, with −7.3 kcal/mol affinity. There were 4 combination points of molecular docking between quercetin and AKR1B1.

## Discussion

*Helicobacter pylori* infection could cause a variety of digestive diseases, such as chronic gastritis, gastric ulcer, duodenal ulcer, gastroesophageal reflux, atrophic gastritis, and GC (8, 9, 29–31). The prevalence of HPI is still a serious concern. According to a systematic review with meta-analysis that involved 410,879 participants from 73 countries in 6 continents, the prevalence of HPI was 44.3% (95% CI: 40.9–47.7) worldwide, ranging from 50.8% (95% CI: 46.8–54.7) in developing countries to 34.7% (95% CI: 30.2–39.3) in developed countries (32). Another published research conducted in Hangzhou, China, described that the positive rates of HPI increased with age (χ^2^ = 116.002, *p* < 0.01) and were 14.8, 20.2, and 25.8% in 3–6, 7–11, and 12–17 years age group, respectively (33). The prevalence of HPI among all population, children, and adults in Iran were estimated as 54% (53–55%), 42% (41–44%), and 62% (61–64%), respectively (34). HP can mainly cause gastritis and gastrointestinal ulcer, and the infected patients would generally have increased gastric secretion, and a few could further lead to atrophic gastritis, or even lymphoma and GC in gastric mucosa associated lymphoid tissue (35).

The findings of this study revealed that the relationship between HPI and AST was involved in the process of response to bacterium and regulated exocytosis, having the regulatory effect for the key targets, such as CXCL9, CCL20, CCL4, etc.

It was shown that CXCL9, which is the ligand with the lowest affinity for CXCR3, has the function of regulating immune cell migration, differentiation, and activation, with contribution to tumor suppression (36, 37), which accounts for the suppression of CXCL9/CXCR3 on GC. The study by Tokunaga et al. suggested that the expression of CXCL9 among patients with HPI was upregulated (37). CXCL9/CXCR3 can chemotaxis and recruit macrophages and T cells to the site of infection during the invasion of HP (38). When HPI was suppressed, memory T cells that could express CXCR3 remained. In patients with AST, exposure to bacteria might result in age-dependent sensitization and AST reactions. On the one hand, we hypothesized that when patients with AST were exposed to fungi again, the abovementioned memory T cells were activated rapidly to produce CXCR3, which could be in combination with CXCL9, that inhibited the fungus. On the other hand, it was reported that the severe AST was associated with a larger number of Th2 subgroups and a smaller number of Th1 subgroups. The result of some clinical or animal experiments presented that the serum CXCL9 concentrations were significantly higher in patients with AST than in the healthy group (39–41). The upregulated expression could help reconstruct the balance of Th1/Th1, which could suppress AST (42).

*P53* is an important tumor suppressor gene, which is located in the region of chromosome 17p13.1 and contains 11 exons. It encodes a 53-kD protein, tumor suppressor protein TP53, which contains 393 amino acid residues. The TP53 protein is an important nuclear transcription factor in the human cells, which is involved in regulating cell cycle and apoptosis, maintaining the stability of various gene expressions, regulating the cell growth, differentiation, and aging (43). It was found that TP53 was one of the top 10 key targets in this study, occurring in the influence of HPI on GC, which was in agreement with the previous studies. The study by Ha et al. suggested that a synergistic interaction between HPI and TP53 might play a significant role in the pathogenesis of GC in the Vietnamese population (44). The animal experiment by Shimizu et al. showed that gastric tumors and tissues from the humans and mice indicated that TP53 genetic mutation appeared in 44.1% tissues (45). The result of the KEGG pathway also confirmed that the influence of HPI on GC consist of p53 signaling pathway, multiple cancer signaling pathways, multiple cancer signaling pathways, and PI3K-Akt signaling pathways (46).

The VCAM-1 overexpression was associated with angiogenesis and metastasis in GC, in which both local expression in gastric tissue and serum levels are directly associated with poor prognosis (47). In children, it was described that the serum concentrations of VCAM-1 were higher in symptomatic children with HPI-associated gastritis compared to non-infected children and confirmed that the serum levels of VCAM-1 correlated with HI-induced gastric inflammation and damage (48). It was suggested that VCAM-1 was newly synthesized prior to spontaneous AST attacks (49), and its expression might play a key role in eosinophil infiltration into the airway.

The molecular docking has been widely used to predict the relationship between ingredients/drug and protein/targets (50, 51). In this article, the gradients (quercetin) of *C. chinensis* were operated to dock with targets (AKR1B1 and VCAM1). Quercetin is a flavonoid commonly found in many edible and medicinal plants, such as onions, tea leaves, and *C. chinensis*. As mentioned in the literature reported, it has antioxidation, antitumor, hypoglycemia, blood lipids, and other pharmacological effects (52). A recent research found that quercetin has the anti-allergic properties characterized by stimulation of immune system, inhibition of histamine release, decrease in proinflammatory cytokines, and improvement in the Th1/Th2 balance (53). The experiment by Zhang et al. showed that quercetin protected against gastric inflammation and apoptosis associated with HPI by affecting the levels of p38MAPK, BCL-2, and BAX (54). Thus, quercetin could act as an effective ingredient to protect against HPI and activate some pathways.

## Limitation

The limitation of study was that only GC was selected as one typical digestive disease influenced severely by HPI; in further studies, it will be investigated whether the mechanism of HPI leads to gastric disease synchronously, and how to activate the pathway which protects against AST. Another limitation was that it was predicted abstractly in this study and was not put into animal experimentation; however, the molecular docking could make up for this defect to some extent.

## Conclusion

CXCL9 and VCAM1 were the common targets of AST and HPI, which might be an imported target of HPI for AST. Quercetin could be an effective ingredient to suppress HPI and help prevent AST.

## Data Availability Statement

All data involved in this study can be obtained from the public database.

## Ethics Statement

All procedures performed in the studies involving human participants were in accordance with the ethical standards of the institutional and/or national research committee and with the 1964 Declaration of Helsinki and its later amendments or comparable ethical standards.

## Author Contributions

FW was the guarantor of integrity of the entire study and designed the study. CC and FW dealt with study concepts. CC and FW defined the intellectual content of the manuscript. CC, FP, and FW involved in the literature research and revised and reviewed the manuscript. FP handled data acquisition. CC contributed to data analysis/interpretation, statistical analysis, and manuscript preparation. FW and FP edited the manuscript. All authors participated in this study and consent to publish this article in Frontiers in Oncology.

## Conflict of Interest

The authors declare that the research was conducted in the absence of any commercial or financial relationships that could be construed as a potential conflict of interest.

## Abbreviations

HP: *Helicobacter pylori*
HPI: *Helicobacter pylori* infection
GC: gastric cancer
AST: asthma
GO: gene ontology
KEGG: Kyoto Encyclopedia of Genes and Genomes
PPI: protein–protein interaction
OR: odds risk
CR: coptidis rhizoma
Df: degrees of freedom.
